# A MALDI-TOF MS Approach for Mammalian, Human, and Formula Milks’ Profiling

**DOI:** 10.3390/nu10091238

**Published:** 2018-09-05

**Authors:** Laura Di Francesco, Francesco Di Girolamo, Maurizio Mennini, Andrea Masotti, Guglielmo Salvatori, Giuliano Rigon, Fabrizio Signore, Emanuela Pietrantoni, Margherita Scapaticci, Isabella Lante, Bianca Maria Goffredo, Oscar Mazzina, Ahmed Ibrahim Elbousify, Paola Roncada, Andrea Dotta, Alessandro Fiocchi, Lorenza Putignani

**Affiliations:** 1 Unit of Human Microbiome, Bambino Gesù Children’s Hospital, IRCCS, V.le San Paolo 15, 00146 Rome, Italy; lau.difra@gmail.com (L.D.F.); di_girolamo.f@hotmail.it (F.D.G.); 2 Allergy Unit, Bambino Gesù Children’s Hospital, IRCCS, Piazza Sant’Onofrio 4, 00165 Rome, Italy; maurizio.mennini@opbg.net (M.M.); oscar.mazzina@gmail.com (O.M.); agiovanni.fiocchi@opbg.net (A.F.); 3 Gene Expression-Microarrays Laboratory, Bambino Gesù Children’s Hospital, IRCCS, V.le San Paolo 15, 00146 Rome, Italy; andrea.masotti@opbg.net; 4 Neonatal Intensive Care Unit, Department of Medical and Surgical Neonatology, Bambino Gesù Children’s Hospital, IRCCS, Piazza Sant’Onofrio 4, 00165 Rome, Italy; guglielmo.salvatori@opbg.net (G.S.); andrea.dotta@opbg.net (A.D.); 5 Department of Obstetrics and Gynecology, San Camillo Forlanini Hospital, Circonvallazione Gianicolense 87, 00151 Rome, Italy; rigon.giuliano@gmail.com; 6 Department of Obstetrics and Gynecology, Misericordia Hospital Grosseto, Usl Toscana Sud-est, 58036 Grosseto, Italy; fabrizio.signore@uslsudest.toscana.it; 7 Rehabilitation Hospital of High Specialization of Motta di Livenza, 31100 Treviso, Italy; emanuela.pietrantoni@gmail.com; 8 Department of Laboratory Medicine, San Camillo Hospital, V.le Vittorio Veneto 18, 31100 Treviso, Italy; scapaticci.m@gmail.com (M.S.); Isa.Lante@gmail.com (I.L.); 9 Metabolic Unit, Department of Pediatric Medicine, Bambino Gesù Children’s Hospital, IRCCS, V.le San Paolo 15, 00146 Rome, Italy; biancamaria.goffredo@opbg.net; 10 Tripoli Children Hospital, University of Tripoli, 13019 Tripoli, Libya; elbousify@gmail.com; 11 Department of Health Sciences, Università degli Studi “Magna Græcia” di Catanzaro, 88100 Catanzaro, Italy; roncada@unicz.it; 12 Unit of Parasitology, Bambino Gesù Children’s Hospital, IRCCS, Piazza Sant’Onofrio 4, 00165 Rome, Italy

**Keywords:** infant nutrition, breast milk, mammalian milk, formula milk, protein similarity profiling, MALDI-TOF mass spectrometry

## Abstract

Human milk composition is dynamic, and substitute formulae are intended to mimic its protein content. The purpose of this study was to investigate the potentiality of matrix-assisted laser desorption/ionization-time-of-flight mass spectrometry (MALDI-TOF MS), followed by multivariate data analyses as a tool to analyze the peptide profiles of mammalian, human, and formula milks. Breast milk samples from women at different lactation stages (2 (*n* = 5), 30 (*n* = 6), 60 (*n* = 5), and 90 (*n* = 4) days postpartum), and milk from donkeys (*n* = 4), cows (*n* = 4), buffaloes (*n* = 7), goats (*n* = 4), ewes (*n* = 5), and camels (*n* = 2) were collected. Different brands (*n* = 4) of infant formulae were also analyzed. Protein content (<30 kDa) was analyzed by MS, and data were exported for statistical elaborations. The mass spectra for each milk closely clustered together, whereas different milk samples resulted in well-separated mass spectra. Human samples formed a cluster in which colostrum constituted a well-defined subcluster. None of the milk formulae correlated with animal or human milk, although they were specifically characterized and correlated well with each other. These findings propose MALDI-TOF MS milk profiling as an analytical tool to discriminate, in a blinded way, different milk types. As each formula has a distinct specificity, shifting a baby from one to another formula implies a specific proteomic exposure. These profiles may assist in milk proteomics for easiness of use and minimization of costs, suggesting that the MALDI-TOF MS pipelines may be useful for not only milk adulteration assessments but also for the characterization of banked milk specimens in pediatric clinical settings.

## 1. Introduction

Breast milk is the primary food source for newborn mammals, and the World Health Organization recommends that infants should be exclusively breastfed for the first six months of life [1,2]. BM synthesis is subtly regulated at a local level [3], and its composition is influenced by several factors such as animal species and genetics, environmental conditions, and animal nutritional status [4]. Human milk (HM) composition varies with gestational age, lactation stage (transition from colostrum to late lactation), within feeds, diurnally, and amongst mothers [5,6]. HM provides proteins, tricalcium phosphate, lipids, vitamins, salt, and lactose; it also contains many hundreds to thousands of distinct bioactive molecules which protect against infection and inflammation and contribute to immune maturation, organ development, and healthy microbial colonization [7].

Despite many campaigns for the promotion of breastfeeding, only 38% of infants in the world are exclusively breastfed [8,9]. When HM becomes unsuitable or inadequate, its ideal substitutions are the infant formulae, defined as “a breast milk substitute specially manufactured to satisfy, by itself, the nutritional requirements of infants during the first months of life up to the introduction of appropriate complementary feeding” [10]. Unlike the dynamic composition of HM, infant formulae are standard products with a composition that is highly regulated by the authorities. To date, the most commonly recommended infant formulae are based on cow milk [11]. The Food and Drugs Administration (FDA) in the US and the European Society for Pediatric Gastroenterology Hepatology and Nutrition (ESPGHAN) in Europe recommended that the formulae should be enriched in whey protein fractions and lowered in caseins [9,12]. The worldwide recommendations are primarily based on the chemical analysis of human milk, and manufacturers are continually modifying their products to make them more similar to human breast milk, the gold standard to estimate the needs of an infant [13], and to increase their health benefits, including iron, nucleotides, prebiotics, and compositions of fat blends [14]. In this context, the number of studies on HM and its protein composition has dramatically increased during the last half century [15,16,17,18,19]. For children with a cow’s milk allergy (CMA) whose mother cannot breastfeed, milk from different mammals has been evaluated, but no milk formulae from animals other than cows has been formulated, and cross-reactivity is possible between the proteins of cows and other mammalian milk [20]. Thus, other animal-milk-based formulae are currently not recommended [21].

The recommendations on substitute formulae in cases of lactation failure are based on many factors, but nutritional considerations are one of the prominent factors. Among the nutritional factors, proteins are the most important. This study aims to investigate the potentiality of linear matrix-assisted laser desorption/ionization time-of-flight mass spectrometry (MALDI-TOF MS) as a tool to assess the diversity and oddness of different artificial and mammalian kinds of milk compared to the reference human milk. MALDI-TOF MS is a platform adopted by many healthcare clinical laboratories worldwide owing to its simplicity of use, high reproducibility of the mass spectra, and low cost of the analysis [22]. Recently, this technique has been proposed as a powerful tool to obtain informative fingerprints of milk proteins [23]. The identification of differences and similarities among several types of artificial and animal milk compared to the reference human milk at different stages of lactation could assist the design of infant formulae. Furthermore, a MALDI-TOF MS-based approach coupled with a multivariate statistical assessment of MS data could represent a versatile workflow to evaluate the quality and safety of sample milk in blind for nonspecialized, mass-spectrometric laboratories.

## 2. Materials and Methods

### 2.1. Milk Sampling and Pre-Treatment

The human milk (HM) samples were collected from twenty healthy breastfeeding mothers at the Department of Obstetrics and Gynecology, San Camillo Forlanini Hospital of Rome, Italy, at four different periods of lactation: 2 days (colostrum, HC), 30 days (HM30), 60 days (HM60), and 90 days (HM90). The study protocol was approved by the Ethics Committee of the San Camillo-Forlanini Hospital (protocol 460/CE; 27/03/2012) and by the Institutional Review Board of the Bambino Gesù Children’s Hospital (protocol 295 LB; 16/05/2012). Informed written consent was obtained from all mothers. Raw donkey milk (DM) from four she-donkeys belonging to the Amiata, Viterbese, and Martina Franca breeds, cow milk (CM) from four cows belonging to the Frisona breed, buffalo milk (BM) from seven buffalos belonging to the Mediterranean Italian breed, goat milk (GM) from four goats belonging to the Maltese breed, and ewe’s milk (EM) from five ewes belonging to the Tuscolania breed were collected from Italian farms (Lazio and Puglia). Camel milk (CAM, from two *Camelus dromedarius*) was collected from Libyan desert farms. Commercially available infant formula milk samples from four different brands were also studied: Aptamil 1 (A) (Mellin SpA, Milan, Italy), Humana 1 (H) (Humana Italia SpA, Milan, Italy), Formulat 1 (F) (Dicofarm, S.A., Rome, Italy), and Nidina 1 (N) (Nestlé, S.A., Milan, Italy). For each brand, we obtained four samples from different batches that were produced over a period of two years. All the animal milk samples were mechanically milked during the middle lactation stage into sterile polystyrene containers, immediately frozen, and stored at −80 °C until use to prevent undesired proteolysis. After thawing, raw milk samples were defatted by a two-step centrifugation using the Eppendorf Centrifuge 5417 R (The LabWorld, Woburn, MA, USA). The first centrifugation was performed at 3000× *g* for 10 min at 4 °C. The skimmed milk was then centrifuged at 20,000× *g* for 20 min at 4 °C to remove bacteria and cell debris. The skimmed milk’s fractions were subsequently diluted 1:100 with ultrapure water (Milli-Q Millipore) and subjected to mass spectrometry analysis [23].

### 2.2. MALDI-TOF Spectra Acquisition

An aliquot (1 μL) of each skimmed milk’s fraction was directly spotted onto an MSP 96 polished steel target (Bruker Daltonics, Bremen, Germany), overlaid with 1 L of matrix, represented by a solution of 10 mg/mL of sinapinic acid (Sigma-Aldrich, St. Louis, MO, USA) in 50% acetonitrile (Sigma-Aldrich, St. Louis, MO, USA), containing 0.1% trifluoroacetic acid (*v*/*v*) (Sigma-Aldrich, St. Louis, MO, USA), and allowed to dry at room temperature. MALDI-TOF analysis was performed with a Microflex LT linear mass spectrometer (Bruker Daltonics, Bremen, Germany) equipped with the FlexControl software package, version 3.0 (Bruker Daltonics, Bremen, Germany), for spectra recording in the positive linear mode (laser frequency 20 Hz; ion source 1 voltage, 20 kV; ion source 2 voltage, 18.4 kV; lens voltage, 9.1 kV; mass range, 2000 Da to 30,000 Da). Four independent spectra (500 shots one step from different positions of the target spot, for spectrum) for each skimmed milk’s fraction were manually collected, externally calibrated by using Bacterial Test Standard (Bruker Daltonics), and subsequently analyzed.

### 2.3. Statistical Analysis 

Before the statistical analysis was conducted, 200 mass spectra, as reported in Table 1, were manually acquired and visually inspected. Subsequently, each spectra was loaded into FlexAnalysis software, version 3.0 (Bruker Daltonics, Bremen, Germany), to perform mass adjustment (spectra were compressed by a factor of 10 in the total mass range), smoothing (mass data were adjusted by the Savitsky–Golay algorithm with a frame size of 25 Da), baseline subtraction (was applied the minimum value for finding the baseline), normalization (was applied the maximum norm to normalize the baseline subtracted data), and peak picking (was applied spectra differentiation algorithm for finding the peaks, maximum peaks 100, threshold 0.1, method Peak Fitting). The total preprocessed raw datasets of the 200 milk spectra were imported into R Bioconductor (http://www.bioconductor.org/) [24] for Pearson’s correlation analysis and hierarchical clustering. The package pvclust was applied for bootstrapping. For each cluster generated by hierarchical computation, *p*-values (between 0 and 1) were calculated via multiscale bootstrap resampling. Two different *p*-values were provided by the package pvclust: approximately unbiased (AU) and bootstrap probability (BP). AU was computed by multiscale bootstrap resampling and represents a better approximation of an unbiased *p*-value than a BP value computed by normal bootstrap resampling. The same preprocessed raw datasets were imported into ClinProToolsTM bioinformatics software, version 2.2 (Bruker Daltonics, Bremen, Germany) [25], and converted into a virtual gel-like format. The mass values (*m*/*z*) were reported on the *X* axis, while the gray scale bar, reported on the *Y* axis, showed the relationship between the color intensity and the peak intensity. Finally, principal component analysis (PCA) was performed via ClinProToolsTM software, version 2.2 (Bruker Daltonics, Bremen, Germany), which employs only the statistically significant peaks after group classification for the calculation. Based on a Welch’s *t*-test, a *p*-value for each peak was calculated. This value indicates the probability that the observed intensity differences among the various peaks are due to chance. These calculations have been done independently for peak heights and peak areas. 

## 3. Results

### 3.1. Low-Molecular-Weight Protein Profiles from Crude Milk by MALDI-TOF MS

**Figure 1:**
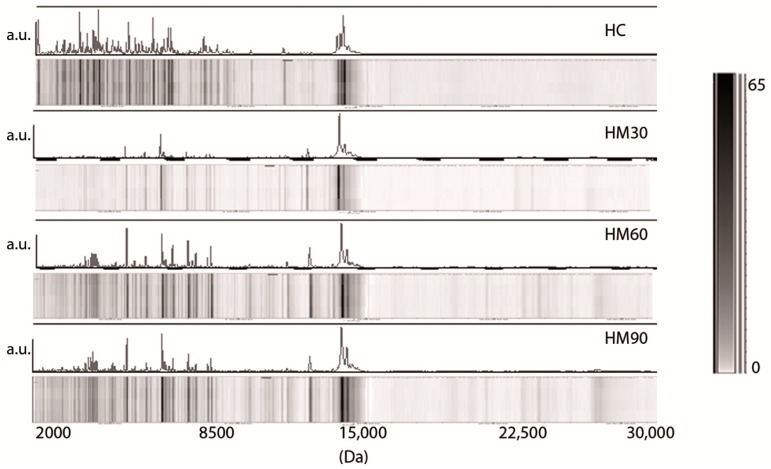
Representative matrix-assisted laser desorption/ionization-time-of-flight mass spectrometry (MALDI-TOF MS) profiling and pseudogel view of crude human milk at two, 30, 60, and 90 days of lactation, indicated respectively as HC, HM30, HM60, and HM90. The mass-to-change ratios (*m*/*z*) are reported on the *X* axis (Da), while the peak intensities are indicated as arbitrary units (a.u.) in the gray scale bar on the *Y* axis.

An analysis of peptides and low-molecular-weight proteins (2000–30,000 Da) present in skimmed raw milk was performed by a benchtop linear MALDI-TOF mass spectrometer. We obtained complex mass spectra that were not affected by signal background problems. Four independent MALDI-TOF MS protein profiles from each milk sample were recorded in order to ascertain a high level of analytical reproducibility for the analysis. Mass spectra obtained from different milk samples from the same source prepared and run on the same day were virtually indistinguishable, and the relative intensities of protein species detected in each replicate were constant. Each spectrum was visually inspected, and the resulting flattened profiles were compared by gel-like representations with spectra from different samples. Figure 1 reports the MALDI-TOF MS profile and the pseudogel view of human milk samples analyzed at 2 (colostrum, HC), 30 (HM30), 60 (HM60), and 90 (HM90) days postpartum. In all human samples, many peaks are visible in the left part of the mass spectrum. After the conversion of the obtained mass spectra to a gel-like format, it appeared clear that many species below a molecular weight of 5000 Da were present especially in the HC samples. In the middle of the spectra (medium-mass range), many peaks between 8500 and 16,000 Da were present in all samples, whereas no peaks were detected in the rightmost part of the spectra (>16,000 Da), although the sinapinic acid matrix, which is beneficial for the ionization of higher molecular weight proteins, was used during the sample deposition on the MALDI target. It was only in mature milk (HM60 and HM90) that a mass value of approximately 24,000 Da became detectable.

**Figure 2:**
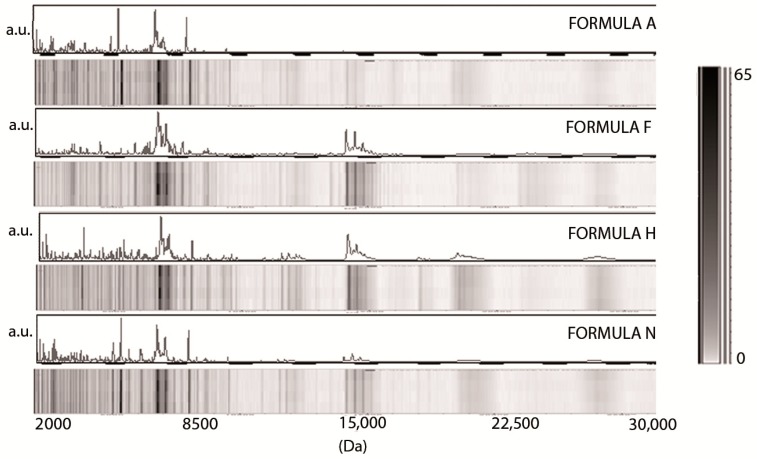
Representative MALDI-TOF MS profiling and pseudogel view of commercial starting formula from four different companies (Aptamil 1, A; Formulat 1 from Dicofarm, F; Humana 1, H; Nidina 1 from Nestlé, N). The mass-to-change ratios (*m*/*z*) are reported on the *X* axis (Da), while the peak intensities are indicated as arbitrary units (a.u.) in the gray scale bar on the *Y* axis.

Figure 2 shows the MALDI-TOF MS profile and the pseudogel view relative to four commercial infant formulas (A, H, F and N). The distribution of molecular weights observed in the mass range between 2000 and 30,000 Da was similar in formula F, H, and N, while formula A displayed peaks only in the first part of the spectra, and no detectable signals were observed in the high-mass range, although it was not a hydrolysate formula. A faint signal above background at approximately *m*/*z* 18,000 was detectable for both formula H and N.

**Figure 3:**
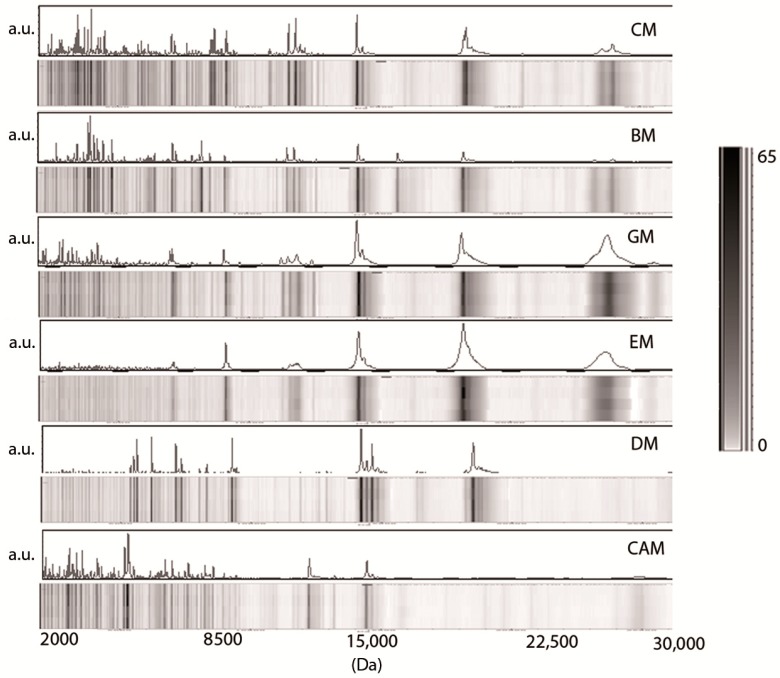
Representative MALDI TOF MS profiling and pseudogel view of crude milk from cow milk (CM), buffalo milk (BM), goat milk (GM), ewe milk (EM), donkey milk (DM), and camel milk (CAM). The mass-to-change ratios (*m*/*z*) are reported on the *X* axis (Da), while the peak intensities are indicated as arbitrary units (a.u.) in the gray scale bar on the *Y* axis.

Figure 3 reports the mass spectra and the relative pseudogel view of other mammalian milk analyzed in the present study: cow milk (CM), buffalo milk (BM), goat milk (GM), ewe milk (EM), donkey milk (DM), and camel milk (CAM). All animal milk samples contain several peaks in the low-, medium- and high-mass range.

In the region of the mass spectra <10,000 Da, many spectrometric signals were detectable except from EM. As in human and formula milk, a mass value of approximately 14,000 Da predominated in all animal samples. Conversely, the mass spectra of animal milk samples showed two intense peaks at approximately 18,000 (missing in human samples as well as CAM) and 24,000 Da (missing in HC, HM30 as well as in DM and CAM). In our conditions, CAM milk showed a profile displaying a reduced number of peaks in the high-mass range compared to other milk.

### 3.2. Milk Spectra Properties and Similarities

To evaluate the similarities and differences among peptide and protein compositions of different milk samples, we used an external statistical software (R Bioconductor) to perform a correlation analysis on spectral values (*m*/*z* and intensities) extracted after ClinProToolsTM bioinformatics software preprocessing.

**Figure 4:**
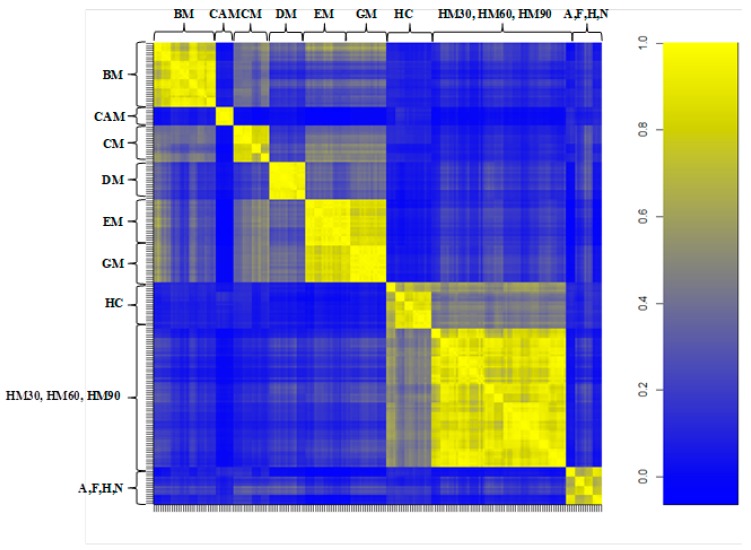
Pearson’s correlation matrix of all spectral replica datasets for animal milk (BM, CAM, CM, DM, EM, and GM), human milk at 2, 30, 60 and 90 days (HC, HM30, HM60 and HM90, respectively), and infant formula (A, F, H, N). Correlation coefficients are represented with decreasing blue and yellow colors according to a scale ranging from 0 to 1, respectively.

Figure 4 displays the correlation matrix obtained from all of the spectra. The figure represents three wide subgroups: the first group of animal milk (BM, CAM, CM, DM, EM, and GM), the second group of human milk (HC, HM30, HM60, and HM90), and the third group of formula milk (A, F, H, and N). From a visual analysis, animal milk does not have an appreciable correlation with human or formula milk. CAM displayed a very poor correlation with all of the considered milk. This may suggest that this milk might have a different protein profile compared to other animal milk. BM has a relatively strong correlation with CM and to a lesser extent with EM and GM, which is in agreement with our previous study [26]. Human colostrum has a good correlation with mature human milk, but HM30, HM60, and HM90 are more correlated between them. Of note, although well correlated with each other, none of the milk formulae analyzed in this study displayed an appreciable correlation with animal or human milk.

**Figure 5:**
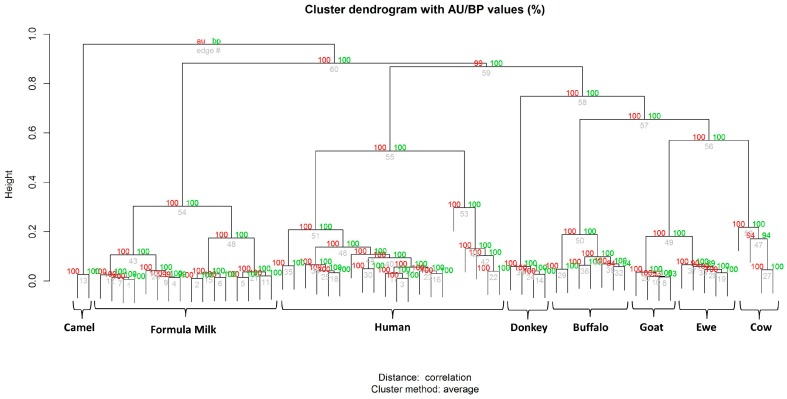
Hierarchical clustering tree (bootstrap *n* = 1000) of all MALDI-TOF spectral replica from animal milk (CM, BM, GM, EM, DM, and CAM), human milk at 2, 30, 60 and 90 days (HC, HM30, HM60 and HM90, respectively), and infant formula (A, F, H, N). Red values (left) are approximately unbiased (AU) *p*-values, green values (right) are bootstrap probability (BP) values, and grey values are cluster labels (bottom).

**Figure 6:**
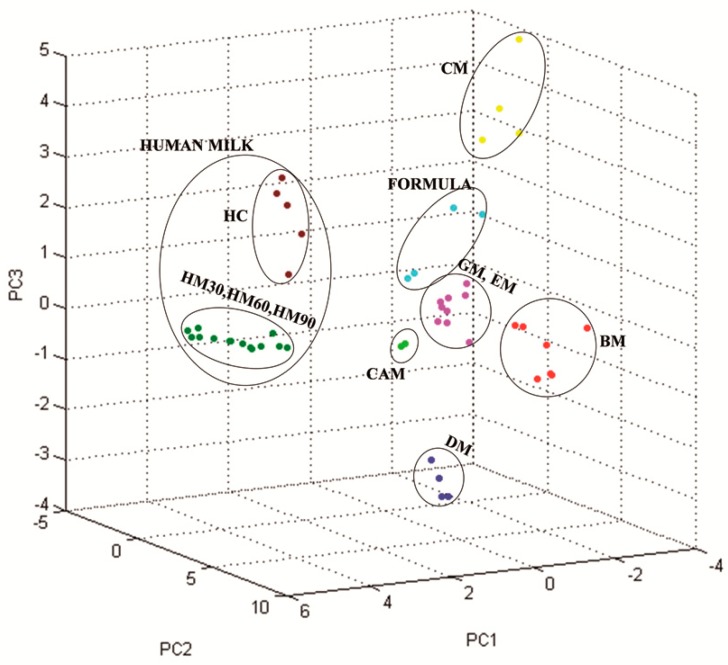
3D scatter plot image from the principal component analysis (PCA) for human milk at 2, 30, 60, and 90 days (HC, HM30, HM60, and HM90, respectively), infant formula milk (A, F, H, and N), and animal milk (CM, BM, GM, EM, DM and CAM). Each spot represents one milk sample.

These considerations were also supported by the hierarchical clustering tree (Figure 5) determined by the statistical software, R Bioconductor. The camel milk cluster separated from the other three clades that represent the group of formula milk, animal milk, and human milk. Formula milk from four different companies displayed a relatively homogeneous clustering, suggesting moderately common spectral characteristics. However, differences among the different brands allowed us to identify a common subcluster for each brand, even across its different batches. The group of human mature milk clustered near the colostrum clade, whereas the animal milk group was relatively well separated. The spectra were then analyzed by principal component analysis (PCA) using the integrated software ClinProTools^TM^. As shown in Figure 6, the milk samples of the same species closely clustered together, whereas the different milk species were well separated each other. The 3D scatter plot image obtained from the PCA analysis indicates that seven MALDI-TOF MS profiles can be grouped, again corresponding to breast milk (with the two subgroups of colostrum and the other stages of breast milk), starting formulae, CM, BM, GM/EM, DM, and CAM.

## 4. Discussion

In the current study, we focused our interest on protein content that serves diverse biological activities in milk such as providing essential amino acids to growing infants, supplying newborns with enzymatic activity, and making available vitamins and hormones. Proteins are present in milk with a very large dynamic range in their concentrations [27]. After a defatting operation consisting in a two-step centrifugation, the milk fat globule membrane proteins were lost in great part together with their lipid counterpart, and the protein content of all samples resulted principally in caseins and soluble whey proteins. We then conducted a qualitative MALDI-TOF MS-based analysis of defatted milk to evaluate the similarities and differences in low-molecular-weight profiles of the composition of milk from a different source.

Multiple components were detected as clear signals in the mass range of 2000–30,000 Da. This region is known to represent milk proteins and peptides with pivotal roles in infants’ health and development such as antimicrobial activities (e.g., lysozyme, 16 kDa and lactalbumin, 14 kDa) and mineral absorption functions (e.g., caseins, between 20 and 25 kDa). Furthermore, most of the bioactive factors are peptides originally present in milk, which may exert their biological activity in the upper gastrointestinal tract regardless of digestive processes [19].

Our data indicate that the human colostrum profile (<30,000 Da) is more complex than every other kind of milk (Figure 1, Figure 2 and Figure 3). Colostrum, secreted in the few days after birth, is reported to contain higher amount of peptides, proteins, and vitamins compared to mature milk [28]. The unique characteristics of HC, with additional nutrients and immune and growth factors, make it interesting as a therapy to promote neonatal health [28,29]. We found that HC spectra are particularly rich in low-molecular-weight proteins and are dominated by spectrometric signals with a mass <10,000 Da and mainly <7000 Da, a significant fraction of which may be involved in its physiological characteristics. Based on the linear MALDI-TOF MS, our analysis does not allow us to identify any milk proteins, but it clearly indicates that the milk protein profile changes gradually over the following 30 days after birth. The amount of peptides and proteins decreases rapidly during the first month of lactation and then stabilizes in “mature” milk after 60 days (HM60 and HM90; Figure 1). By contrast, polypeptides of approximately 15,000 Da remain stable across colostrum and mature milk. A noteworthy finding was that the appearance of spectrometric signals with an *m/z* value of approximately 24,000 Da in mature milk (HM60 and HM90) was concomitant to a decrease in components with a molecular weight <12,000 Da. In agreement with these data, proteomic studies have shed light on the dynamic composition of human milk throughout lactation stages [18,30,31,32]. In particular, whey proteins implicated in the modulation of the immune system and in the maturation of the gastrointestinal tract of neonates are overrepresented in the human milk during the first days of lactation.

A hierarchical cluster analysis of the mass spectra was used to group milk samples according to the similarity of their spectral profiles. In this unsupervised analysis, the group assignment of the protein/peptides expression patterns was generated based on the similarities of spectral patterns in the automatic selected peaks. This analysis demonstrated that all analyzed milk samples formed four main clusters (Figure 5). All human samples (*n* = 20) formed a cluster in which milk at 30, 60, and 90 days constituted a well-defined subcluster. These results indicate that colostrum could be clearly differentiated by signal patterns of their MALDI-TOF mass spectra as an out-group. Even more clearly, the PCA of the qualitative characteristics generated seven principal components (PCs) (Figure 6). Human milk was segregated into a single PC, in which HC (brown dots) was identified as different from human milk samples at different stages of lactation (green dots).

The spectra relative to the commercial starting formulae reported in Figure 2 were from four different brands in which bovine milk is the only source of protein (e.g., casein alone, whey proteins together with caseins, etc.) while fat content was derived from a mixture of vegetable oil. Each group included four different samples of the same formula, coming from different batches. The linear MALDI-TOF MS technique was able to identify a consistent similarity among the profiles of different milk samples. All formulae displayed a profile consistently different from cow’s milk, which is their parent protein source. This is consistent with the ESPGHAN recommendations for the protein composition of infant formulae (12), which were modified such as the formulae studied were all enriched in whey protein fractions and lowered in caseins. However, each brand was characterized by a specific protein profile. Although all 16 samples of infant formula formed a tight cluster (Figure 5), each brand could be differentiated, indicating that the proteomic asset of each formula was stable across batches and could be identified in a blinded, unsupervised analysis.

The animal milk analyzed in the present study showed similar protein peak profiles ranging from 14,000 to 18,000 Da (Figure 3). In particular, these spectrometric signals were conserved across CM, BM, GM, EM, and DM but were less evident in CAM. The spectrometric signals at 18,000 Da and 24,000 Da correspond, respectively, to the theoretical molecular weights of lactoglobulin and casein—two components of cow milk responsible for cow’s milk allergy (CMA), the most common food allergy affecting children [33]. The absence of these spectrometric signals in the CAM sample and in all analyzed human milk is in agreement with previous studies that report the lack of these proteins in camel milk [34,35]. For this reason, CAM, although not extensively used, was recommended by pediatricians for children with a cow’s milk allergy and hence was evaluated in the present work [34,36,37,38,39].

GM and EM clearly displayed two additional peaks at 20,000 and 25,000 Da, which was less evident in CM and BM. They were absent in donkey milk, which was characterized by a notoriously low protein content (1.3–2.8 g/100 mL) and by a high whey protein/casein ratio [40]. The hierarchical cluster analysis of peak profiles indicated that 24 samples of five types of animal’s milk could be subcategorized by source: a subcluster identified DM vs. bovidae milk (buffalo, goat, ewe, cow), another included all seven BM samples, and a third was composed by the 13 samples of CM, EM, and GM. The results indicate that cow, ewe, and goat milk have a homogeneous milk proteome, while milk proteins from donkey and cow milk share a low-sequence similarity due to the genetic distance between the *Equidae* and *Bovidae* families. Indeed, there is evidence of cross-reactivity between cow milk and proteins from goat, sheep and buffalo milk [20], while substantial differences in the IgE-binding epitope of cow milk proteins and the corresponding domains of donkey milk protein may, besides the low content in caseins, account for the demonstrated reduced allergenicity of DM [41]. Its distinctive protein composition is also evident in the results of our PCA, where we were able to differentiate between BM and CM samples and also between these ones and DM. The only two kinds of milk that clustered together were from ewe and goat samples, which was not a surprising finding if we consider the taxonomic proximity between goats and sheep (i.e., *Caprinae* subfamily) and the well-known clinical cross-reactivity among their milk [42]. Conversely, camel milk is completely different from any other mammalian milk from a proteomic point of view. The same observation stems from the PCA reported in Figure 6, where CAM is segregated with a different color. These results confirm the moderately different protein composition of camel milk compared to other animals’ milk [43]. Among the mammalian species that are proposed to be suitable as a valid substitute of cow’s milk-based formulas, the CAM has a unique spectra profile that could have interesting properties in the nutrition of children.

These findings seem to suggest that the choice of an alternative to breast milk cannot be made exclusively on the basis of macronutrient composition, but that the proteomic profile can be a useful evaluation tool [44,45]. Their results may be even more relevant if they will be replicated in extensively hydrolyzed milk formulae (eHFs). These formulae are the first-choice milk substitutes in CMA, but may carry residual allergens that are able to cause reactions in sensitive infants [21]. A recent proteomic analysis revealed that the peptide profiles of commercially available eHFs also provide a descriptive and distinct signature [46].

## 5. Conclusions

MALDI-TOF MS profiling of milk proteins in combination with statistic tools proved to be a high throughput and low-cost approach with promising applications as an analytical tool to quickly assess the similarities and differences of low-molecular-weight proteins present in milk from different sources with a high level of accuracy and sensitivity. Our data point to differences between the potential alternative sources of infant formula milk and create the basis for further proteomic investigations to achieve more conclusive results on the protein content of the milk types herein evaluated.

Moreover, a MALDI-TOF mass spectral database compilation can assist nonspecialized mass spectrometric laboratories for a rapid screening and characterization of milk samples. The screening procedures could become a powerful method for analyzing milk in a blinded way in order to evaluate animal milk adulterations, milk samples present in human donor breast milk banking, and matching between human-milk formula compositions. A rapid MALDI- TOF MS assay could also become an instrument for interpreting the individuality in the phenotypic expression of allergies to cow’s milk proteins and beyond.

## Figures and Tables

**Table 1:** Milk samples analyzed and relative mass spectra acquired.

Milk Source	Samples (*n*)	Spectra Replicates (*n*)	Total Spectra Analyzed (*n*)
Human milk			
Breastfeeding women at 2 days	5	4	20
Breastfeeding women at 30 days	6	4	24
Breastfeeding women at 60 days	5	4	20
Breastfeeding women at 90 days	4	4	16
Commercial brands of infant formula	4	4	16
Animal milk ^1^			
cows	4	4	16
buffaloes	7	4	28
goats	4	4	16
ewes	5	4	20
she-donkeys	4	4	16
camels	2	4	8

^1^ All animal milk samples were collected at middle lactation stage.

## References

[1] Dewey K. (2003). Guiding Principles for Complementary Feeding of the Breastfed Child.

[2] Lessen R., Kavanagh K. (2015). Position of the academy of nutrition and dietetics: Promoting and supporting breastfeeding. J. Acad. Nutr. Diet..

[3] Weaver S.R., Hernandez L.L. (2016). Autocrine-paracrine regulation of the mammary gland1. J. Dairy Sci..

[4] Prentice P., Ong K.K., Schoemaker M.H., van Tol E.A.F., Vervoort J., Hughes I.A., Acerini C.L., Dunger D.B. (2016). Breast milk nutrient content and infancy growth. Acta Paediatr..

[5] Ballard O., Morrow A.L. (2013). Human milk composition. Pediatr. Clin. North Am..

[6] Roncada P., Stipetic L.H., Bonizzi L., Burchmore R.J.S., Kennedy M.W. (2013). Proteomics as a tool to explore human milk in health and disease. J. Proteom..

[7] Del Chierico F., Vernocchi P., Petrucca A., Paci P., Fuentes S., Praticò G., Capuani G., Masotti A., Reddel S., Russo A. (2015). Phylogenetic and Metabolic Tracking of Gut Microbiota during Perinatal Development. PLoS ONE.

[8] Cai X., Wardlaw T., Brown D.W. (2012). Global trends in exclusive breastfeeding. Int. Breastfeed. J..

[9] Demonstration of the Quality Factor Requirements Under 21 CFR 106.96(i) for “Eligible” Infant Formulas. https://www.fda.gov/RegulatoryInformation/Guidances/ucm400036.htm.

[10] Food and Agriculture Organization of the United Nations World Health Organization CODEX alimentarius: Standard for Infant Formula and Formulas for Special Medical Purposes Intended for Infants. http://www.fao.org/fao-who-codexalimentarius/sh-proxy/pt/?lnk=1&url=https%253A%252F%252Fworkspace.fao.org%252Fsites%252Fcodex%252FMeetings%252FCX-720-38%252FReport%252FFINAL%252FREP17_NFSDUe.pdf.

[11] Rossen L.M., Simon A.E., Herrick K.A. (2016). Types of infant formulas consumed in the united states. Clin. Pediatr. (Phila.).

[12] Koletzko B., Baker S., Cleghorn G., Neto U.F., Gopalan S., Hernell O., Hock Q.S., Jirapinyo P., Lonnerdal B., Pencharz P. (2005). Global standard for the composition of infant formula: Recommendations of an ESPGHAN coordinated international expert group. J. Pediatr. Gastroenterol. Nutr..

[13] Stam J., Sauer P.J., Boehm G. (2013). Can we define an infant’s need from the composition of human milk?. Am. J. Clin. Nutr..

[14] Ryan A.S., Hay W.W. (2015). Challenges of infant nutrition research: A commentary. Nutr. J..

[15] Gura T. (2014). Nature’s first functional food. Science.

[16] Weber M., Grote V., Closa-Monasterolo R., Escribano J., Langhendries J.P., Dain E., Giovannini M., Verduci E., Gruszfeld D., Socha P. (2014). Lower protein content in infant formula reduces BMI and obesity risk at school age: Follow-up of a randomized trial. Am. J. Clin. Nutr..

[17] Hidayat K., Du H.-Z., Yang J., Chen G.C., Zhang Z., Li Z.N., Qin L.Q. (2017). Effects of milk proteins on blood pressure: A meta-analysis of randomized control trials. Hypertens. Res..

[18] Gao X., McMahon R.J., Woo J.G., Davidson B.S., Morrow A.L., Zhang Q. (2012). Temporal changes in milk proteomes reveal developing milk functions. J. Proteome Res..

[19] Savino F., Sorrenti M., Benetti S., Lupica M.M., Liguori S.A., Oggero R. (2012). Resistin and leptin in breast milk and infants in early life. Early Hum. Dev..

[20] Restani P., Gaiaschi A., Plebani A., Beretta B., Cavagni G., Fiocchi A., Poiesi C., Velonà T., Ugazio A.G., Galli C.L. (1999). Cross-reactivity between milk proteins from different animal species. Clin. Exp. Allergy J. Br. Soc. Allergy Clin. Immunol..

[21] Fiocchi A., Brozek J., Schünemann H., Bahna S.L., von Berg A., Beyer K., Bozzola M., Bradsher J., Compalati E., Ebisawa M. (2010). World Allergy Organization (WAO) Special Committee on Food Allergy World Allergy Organization (WAO) Diagnosis and Rationale for Action against Cow’s Milk Allergy (DRACMA) Guidelines. Pediatr. Allergy Immunol..

[22] Putignani L., Del Chierico F., Onori M., Mancinelli L., Argentieri M., Bernaschi P., Coltella L., Lucignano B., Pansani L., Ranno S. (2011). MALDI-TOF mass spectrometry proteomic phenotyping of clinically relevant fungi. Mol. BioSyst..

[23] Di Girolamo F., Masotti A., Salvatori G., Scapaticci M., Muraca M., Putignani L. (2014). A sensitive and effective proteomic approach to identify she-donkey’s and goat’s milk adulterations by MALDI-TOF MS Fingerprinting. Int. J. Mol. Sci..

[24] Gentleman R.C., Carey V.J., Bates D.M., Bolstad B., Dettling M., Dudoit S., Ellis B., Gautier L., Ge Y., Gentry J. (2004). Bioconductor: Open software development for computational biology and bioinformatics. Genome Biol..

[25] Ketterlinus R., Hsieh S.-Y., Teng S.-H., Lee H., Pusch W. (2005). Fishing for biomarkers: Analyzing mass spectrometry data with the new ClinProTools software. BioTechniques.

[26] Di Girolamo F., D’Amato A., Lante I., Signore F., Muraca M., Putignani L. (2014). Farm animal serum proteomics and impact on human health. Int. J. Mol. Sci..

[27] Bislev S.L., Deutsch E.W., Sun Z., Farrah T., Aebersold R., Moritz R.L., Bendixen E., Codrea M.C. (2012). A Bovine Peptide Atlas of milk and mammary gland proteomes. Proteomics.

[28] Gephart S.M., Weller M. (2014). Colostrum as Oral Immune Therapy to Promote Neonatal Health. Adv. Neonatal Care.

[29] Bagwe S., Tharappel L.J.P., Kaur G., Buttar H.S. (2015). Bovine colostrum: An emerging nutraceutical. J. Complement. Integr. Med..

[30] Liao Y., Alvarado R., Phinney B., Lönnerdal B. (2011). Proteomic characterization of human milk whey proteins during a twelve-month lactation period. J. Proteome Res..

[31] Liao Y., Alvarado R., Phinney B., Lönnerdal B. (2011). Proteomic characterization of human milk fat globule membrane proteins during a 12 month lactation period. J. Proteome Res..

[32] Reinhardt T.A., Lippolis J.D. (2008). Developmental Changes in the milk fat globule membrane proteome during the transition from colostrum to milk. J. Dairy Sci..

[33] Sackesen C., Assa’ad A., Baena-Cagnani C., Ebisawa M., Fiocchi A., Heine R.G., Von Berg A., Kalayci O. (2011). Cow’s milk allergy as a global challenge. Curr. Opin. Allergy Clin. Immunol..

[34] Shabo Y., Barzel R., Margoulis M., Yagil R. (2005). Camel milk for food allergies in children. Isr. Med. Assoc. J. IMAJ.

[35] Merin U., Bernstein S., Bloch-Damti A., Yagil R., van Creveld C., Lindner P., Gollop N. (2001). A comparative study of milk serum proteins in camel (Camelus dromedarius) and bovine colostrum. Livest. Prod. Sci..

[36] El-Agamy E.I., Nawar M., Shamsia S.M., Awad S., Haenlein G.F.W. (2009). Are camel milk proteins convenient to the nutrition of cow milk allergic children?. Small Rumin. Res..

[37] El-Agamy E.I. (2007). The challenge of cow milk protein allergy. Small Rumin. Res..

[38] Järvinen K.M., Chatchatee P. (2009). Mammalian milk allergy: Clinical suspicion, cross-reactivities and diagnosis. Curr. Opin. Allergy Clin. Immunol..

[39] Ehlayel M.S., Hazeima K.A., Al-Mesaifri F., Bener A. (2011). Camel milk: An alternative for cow’s milk allergy in children. Allergy Asthma Proc..

[40] Piovesana S., Capriotti A.L., Cavaliere C., La Barbera G., Samperi R., Zenezini Chiozzi R., Laganà A. (2015). Peptidome characterization and bioactivity analysis of donkey milk. J. Proteom..

[41] Gallina S., Cunsolo V., Saletti R., Muccilli V., Di Francesco A., Foti S., Lorenzten A.M., Roepstorff P. (2016). Sequence characterization and glycosylation sites identification of donkey milk lactoferrin by multiple enzyme digestions and mass spectrometry. Amino Acids.

[42] Restani P., Fiocchi A., Beretta B., Velonà T., Giovannini M., Galli C.L. (1997). Meat allergy: III—Proteins involved and cross-reactivity between different animal species. J. Am. Coll. Nutr..

[43] Sabahelkhier M., Faten M., Omer F. (2012). Comparative Determination of biochemical constituents between animals (goat, sheep, cow and camel) milk with human milk. Res. J. Recent Sci..

[44] Roncada P., Piras C., Soggiu A., Turk R., Urbani A., Bonizzi L. (2012). Farm animal milk proteomics. J. Proteom..

[45] D’auria E., Agostoni C., Giovannini M., Riva E., Zetterström R., Fortin R., Greppi G., Bonizzi L., Roncada P. (2005). Proteomic evaluation of milk from different mammalian species as a substitute for breast milk. Acta Paediatr..

[46] Lambers T.T., Gloerich J., van Hoffen E., Alkema W., Hondmann D.H., van Tol E.A.F. (2015). Clustering analyses in peptidomics revealed that peptide profiles of infant formulae are descriptive. Food Sci. Nutr..

